# Vitamin D deficiency aggravates ischemic acute kidney injury in rats

**DOI:** 10.14814/phy2.12331

**Published:** 2015-03-16

**Authors:** Ana Carolina de Bragança, Rildo A Volpini, Daniele Canale, Janaína G Gonçalves, Maria Heloisa M Shimizu, Talita R Sanches, Antonio C Seguro, Lúcia Andrade

**Affiliations:** Division of Nephrology, Laboratory of Basic Science LIM-12, University of São Paulo School of MedicineSão Paulo, Brazil

**Keywords:** Acute kidney injury, vitamin D deficiency, vitamin D receptors, cyclin-dependent kinase inhibitor p21

## Abstract

Vitamin D deficiency (VDD) increases the risk of death in hospitalized patients. Renal ischemia/reperfusion injury (IRI) induces acute kidney injury (AKI), which activates cell cycle inhibitors, including p21, a cyclin-dependent kinase inhibitor and genomic target of 25-hydroxyvitamin D, which is in turn a potent immunomodulator with antiproliferative effects. In this study, we assess the impact of VDD in renal IRI. Wistar rats were divided into groups, each evaluated for 30 days: control (receiving a standard diet); VDD (receiving a vitamin D-free diet); IRI (receiving a standard diet and subjected to 45-min bilateral renal ischemia on day 28); and VDD + IRI (receiving a vitamin D-free diet and subjected to 45-min bilateral renal ischemia on day 28). At 48 h after IRI, animals were euthanized; blood, urine, and kidney tissue samples were collected. Compared with IRI rats, VDD + IRI rats showed a more severe decrease in glomerular filtration rate, greater urinary protein excretion, a higher kidney/body weight ratio and lower renal aquaporin 2 expression, as well as greater morphological damage, characterized by increased interstitial area and tubular necrosis. Our results suggest that the severity of tubular damage in IRI may be associated with downregulation of vitamin D receptors and p21. VDD increases renal inflammation, cell proliferation and cell injury in ischemic AKI.

## Introduction

Vitamin D not only influences bone metabolism but also plays a central role in cell functions such as multiplication, differentiation, and metabolism (Powers and Gilchrest 2012). Vitamin D deficiency (VDD) is a risk factor for infectious, autoimmune, neurodegenerative, and cardiovascular diseases, as well as for diabetes, osteoporosis, and cancer (Dusso et al. 2005). The biologically active form of vitamin D is formed in the kidney by mitochondria of the proximal convoluted tubules, where 1*α*-hydroxylase converts 25-hydroxyvitamin D[25(OH)D] to 1,25-dihydroxyvitamin D_3_, or calcitriol. The classical calcitriol pathway requires the nuclear vitamin D receptor (VDR), which is a transcription factor for calcitriol target genes (Zehnder and Hewison 1999; Hewison et al. 2000; Dusso et al. 2005). Liu et al. (1996) showed that p21 (a cyclin-dependent kinase inhibitor that regulates G1-to-S-phase cell cycle progression) is transcriptionally induced by calcitriol in a VDR-dependent manner, as well as identifying a functional vitamin D response element in the p21 promoter. In uremic patients with secondary hyperparathyroidism, Taniguchi et al. (2006) showed that reduced parathyroid expression of p21 is VDR dependent and is a pathogenic factor in parathyroid gland hyperplasia; VDR expression correlated negatively with proliferative activity of the parathyroid glands. In hyperparathyroidism patients with low VDR expression, reduced expression of p21 and p27 constitute a major pathogenic feature of nodular hyperplasia of parathyroid glands (Tokumoto et al. 2002).

Renal ischemia/reperfusion injury (IRI) continues to be a major cause of acute kidney injury (AKI) (Price et al. 2003; de Araujo et al. 2005; Basile et al. 2012). After AKI, various quiescent, terminally differentiated kidney cells enter the cell cycle, (Megyesi et al. 2001) resulting in rapid induction of p21 (Megyesi et al. 1996). Megyesi et al. (2001) studied the effects of p21 induction in AKI by comparing wild-type p21^+/+^ mice and mice homozygous for a p21 gene deletion; after IRI, the kidney cells of p21^−/−^ mice show higher 5-bromo-2-deoxyuridine incorporation into nuclear DNA and proliferating cell nuclear antigen (PCNA) content than do those of p21^+/+^ mice. The authors found that stressing the kidney caused cells to enter the cell cycle, while also inducing a protein identified as a cell cycle inhibitor, although p21 induction ameliorated AKI (Megyesi et al. 2001). After IRI, there was less morphological damage in p21^+/+^ mice than in p21^−/−^ mice, the latter showing a more rapid onset of AKI and higher mortality (Megyesi et al. 2001).

We hypothesized that VDD can increase the severity of morphological damage in ischemic AKI. Therefore, we examined expressions of VDR, p21, ED1, and CD3, as well as tubular injury and PCNA content, in order to elucidate the pathogenic effects of VDD on rat renal cells in AKI.

## Materials and Methods

### Animals

Male Wistar rats (180–200 g) were provided by the University of São Paulo School of Medicine animal facility. During the 30-day experiment, rats receiving vitamin D-free or standard diets (*MP Biomedicals*, Irvine, CA) and free access to tap water. Rats were divided into four groups: control (receiving a standard diet; *n *=* *7); VDD (receiving a vitamin D-free diet; *n *=* *8); IRI (receiving a standard diet and subjected to bilateral renal ischemia for 45 min on day 28; *n *=* *7); and VDD + IRI (receiving a vitamin D-free diet and subjected to bilateral renal ischemia for 45 min on day 28; *n *=* *8). The Ethics Committee of the University of São Paulo School of Medicine approved the experimental protocol.

### Ischemia/reperfusion

On day 28, rats were anesthetized with 2,2,2-tribromoethanol (250 mg/kg BW). The kidneys were exposed through a midline incision; both renal arteries were clamped for 45 min and released. All studies were performed 48 h after renal ischemia/reperfusion, on day 30.

### Urinary variables

On day 29, the rats were placed in metabolic cages (one per cage), on a 12/12-h light/dark cycle, with free access to drinking water. We collected 24-h urine samples, centrifuged them to remove suspended material and analyzed the supernatants. We measured water intake and urine output. We determined urine osmolality with a freezing-point osmometer (model 3D3; Advanced Instruments, Norwood, MA). Urinary concentrations of calcium, phosphorus, and protein were measured by colorimetry (Labtest Diagnóstica, Lagoa Santa, Brazil). We also determined FECa and FEP.

### Inulin clearance

On day 30, the animals were anesthetized with sodium thiopental (50 mg/kg BW). The trachea was cannulated with a PE-240 catheter, and spontaneous breathing was maintained. The jugular veins were cannulated with PE-60 catheters for infusion of inulin and fluids. To monitor MAP and collect blood samples, the right femoral artery was catheterized with a PE-50 catheter. We assessed MAP with a data acquisition system (MP100; Biopac Systems, Santa Barbara, CA). To collect urine samples, the bladder was cannulated with a PE-240 catheter by suprapubic incision. After the surgical procedure, a loading dose of inulin (100 mg/kg BW diluted in 1 mL of 0.9% saline) was administered through the jugular vein. A constant infusion of inulin (10 mg/kg BW) was started and continued at 0.04 mL/min throughout the experiment. Three urine samples were collected at 30-min intervals. Inulin clearance values represent the mean of the three periods. Blood samples were obtained at the beginning and end of the experiment. Serum and urinary inulin were determined by the anthrone method, and glomerular filtration is expressed as mL/min/100 g BW. To measure renal blood flow (RBF), we made a median incision, dissected the left renal pedicle and isolated the renal artery without disturbing the renal nerves. An ultrasonic flow probe was placed around the exposed renal artery, and RBF was measured (in mL/min) with an ultrasonic flow meter (T402; Transonic Systems, Bethesda, MD). To calculate RVR (in mmHg/mL/min), we divided blood pressure by RBF.

### Biochemical parameters

To assess serum levels of 25(OH)D, PTH, phosphate and ionized calcium, we collected blood samples after the clearance studies. We assessed 25(OH)D by radioimmunoassay (DiaSorin, Stillwater, MN), PTH by enzyme-linked immunosorbent assay (Immutopics, San Clemente, CA), phosphate by colorimetry (Labtest Diagnóstica) and calcium with specific electrodes (ABL800 FLEX; Radiometer, Brønshøj, Denmark).

### Tissue samples

After the clearance experiment, we perfused kidneys with phosphate-buffered solution. Right kidneys were frozen in liquid nitrogen and stored at −80°C for western blotting and real-time quantitative polymerase chain reaction (qPCR). Left kidneys were removed and weighed. To obtain the KW/BW ratio, the kidney weight in grams was divided by the body weight in grams and multiplied by 100. Fragments of left kidneys were fixed in 10% neutral-buffered formalin solution or methacarn solution for 24 h and in 70% alcohol thereafter. Kidney blocks were embedded in paraffin and cut into 4-*μ*m sections for histology and immunohistochemistry.

### Kidney fractions

Kidney samples were homogenized in ice-cold isolation solution (200 mmol/L mannitol, 80 mmol/L HEPES and 41 mmol/L KOH, pH 7.5) containing a protease inhibitor cocktail (Sigma Chemical Company, St. Louis, MO) in a homogenizer (PT 10/35; Brinkmann Instruments, Westbury, NY). Homogenates were centrifuged at 2000 *× g* for 15 min at 4°C to remove nuclei and cell debris. Supernatants were isolated, and protein was quantified by Bradford assay (BioAgency Laboratórios, São Paulo, Brazil).

### Electrophoresis and immunoblotting

Kidney samples were run on polyacrylamide minigels (Burnette 1981). After transfer by electroelution to nitrocellulose membranes (GE Healthcare Limited, Little Chalfont, UK), blots were blocked with 5% nonfat dry milk in Tris-buffered saline solution. Blots were then incubated overnight with antibodies against AQP2 (1:2000; Santa Cruz Biotechnology, Santa Cruz, CA); actin (1:5000; Santa Cruz Biotechnology); p21 (1:500; Santa Cruz Biotechnology); VDR (1:500; Santa Cruz Biotechnology). The labeling was visualized with horseradish peroxidase-conjugated secondary antibody (anti-rabbit IgG, 1:2000, or anti-goat, 1:10000; Sigma Chemical, St. Louis, MO) and enhanced chemiluminescence (ECL) detection (Amersham Pharmacia Biotech, Piscataway, NJ).

### Kidney protein levels

We scanned the ECL films with an imaging system (Alliance 4.2; UVItec, Cambridge, UK). We used densitometry to quantitatively analyze the antibodies, normalizing the bands to actin expression.

### Light microscopy

Four-micrometer histological sections of kidney tissue were stained with hematoxylin–eosin or Masson's trichome and examined under light microscopy. We quantified FIA by analyzing tubulointerstitial involvement. For histomorphometry, the images obtained by microscopy were captured on video via an image analyzer (Axiovision; Carl Zeiss, Eching, Germany). We analyzed 30 grid fields (0.087 mm^2^ each) per kidney cortex. The interstitial areas were demarcated manually, and the proportion of the field they occupied, excluding the glomeruli, was determined. In 40–60 grid fields (0.245 mm^2^ each; magnification, ×400), we graded the proportional renal damage (tubular epithelial swelling, vacuolar degeneration, necrosis, and desquamation): 0, < 5%; I, 5–25%; II, 26–50%; III, 51–75%; and IV, > 75%. To minimize bias in the morphometric analysis, the observer was blinded to the treatment groups. The mean scores were calculated by rat and by group (Miyaji et al. 2001).

### Immunohistochemistry

We used the following antibodies: mouse monoclonal antibody to ED1 (1:100 overnight at 4°C; AbD Serotec, Oxford, UK); monoclonal antibody to PCNA (1:500 for 60 min at 20°C; Sigma Aldrich); monoclonal antibody to CD3 (1:100 for 60 min at 20°C; DAKO, Glostrup, Denmark); and polyclonal antibodies to p21, AQP2, and VDR (1:40, 1:100, and 1:100, respectively, for 60 min at 20°C; Santa Cruz Biotechnology). We subjected 4-*μ*m kidney tissue sections to immunohistochemical reaction according to the protocol for each primary antibody. Reaction products were detected by avidin-biotin-peroxidase (Vector Laboratories, Burlingame, CA), and the color reaction was developed in 3,3-diaminobenzidine (Sigma Chemical) and hydrogen peroxide. Counterstaining was with Harris' hematoxylin. For ED1, PCNA, and CD3, we analyzed 30–50 renal cortex fields. The results of the immunoreactions were quantified by counting the number of positive cells per 0.087-mm^2^ field and averaging the number of cells per field for each section. To evaluate immunoreactivity to p21, the volume ratios of positive areas of renal tissue (in %) (Lancas et al. 2011), determined by the color limit, were obtained by image analysis with the program Image-Pro Plus, version 4.1 (Media Cybernetics, Silver Spring, MD) on a computer coupled to a microscope (Axioskop 40; Carl Zeiss) and a digital camera.

### Gene expression

We performed real-time qPCR in frozen renal tissue, assessing the following gene: VDR (Rn00690616-m1). We extracted and prepared total RNA. For cDNA synthesis, we used total RNA and SuperScript VILO MasterMix (Invitrogen, Carlsbad, CA). We performed real-time PCR using TaqMan (Applied Biosystems, Foster City, CA) on Step One Plus (Applied Biosystems). Primers were purchased from Invitrogen. We evaluated relative gene expression with the 2^−ΔΔCt^ method (Livak and Schmittgen 2001), using glyceraldehyde 3-phosphate dehydrogenase as the housekeeping gene.

### Statistical analysis

All quantitative data are mean ± SEM. We analyzed differences among the means of multiple parameters by one-way analysis of variance followed by the Student-Newman-Keuls test. Values of *P *<* *0.05 were considered statistically significant.

## Results

### Biochemical, renal and hemodynamic parameters

As can be seen in Table1, over the 30-day experimental period, VDD rats (those receiving a vitamin D-free diet) developed low serum levels of 25(OH)D. As expected, IRI rats (those receiving a standard diet and subjected to ischemia of both kidneys for 45 min on day 28) showed decreased renal function (inulin clearance measured at sacrifice, on day 30), higher mean arterial pressure (MAP) and greater renal vascular resistance (RVR) than did control rats (those receiving a standard diet). However, inulin clearance was lower in VDD + IRI rats (those receiving a vitamin D-free diet and subjected to bilateral renal ischemia for 45 min on day 28) than in IRI rats, demonstrating that VDD potentiates ischemia/reperfusion-induced AKI. In addition, urinary protein excretion was markedly higher in VDD + IRI rats than in control rats. There were no differences between IRI rats and VDD + IRI rats regarding MAP or RVR. Compared with control rats, VDD rats showed lower inulin clearance, higher MAP and higher RVR.

**Table 1:** Biochemical and hemodynamic parameters in rats fed a standard or vitamin D-free diet and subjected or not to renal ischemia/reperfusion

Parameter	Group			
	C	VDD	IRI	VDD+IRI
Serum 25(OH)D (ng/mL)	14.79 ± 0.88	4.10 ± 0.79^a^	12.98 ± 1.04^d^	3.84 ± 0.23^a^^,^^g^
InCl (mL/min/100 g BW)	0.93 ± 0.05	0.76 ± 0.03^b^	0.43 ± 0.04^a^^,^^d^	0.29 ± 0.03^a^^,^^d^^,^^i^
BW (g)	389.7 ± 9.4	396.4 ± 9.4	320.1 ± 4.4^a^^,^^d^	337.4 ± 7.0^a^^,^^d^
KW/BW ratio	0.40 ± 0.02	0.41 ± 0.01	0.52 ± 0.01^b^^,^^e^	0.58 ± 0.02^a^^,^^d^^,^^i^
MAP (mmHg)	118.9 ± 4.2	134 ± 3.4^c^	136.5 ± 4.3^b^	139 ± 4.2^c^
RVR (mmHg/mL/min)	21.0 ± 0.7	23.86 ± 0.6^c^	24.49 ± 0.7^b^	24.56 ± 0.7^c^
Serum PTH (pg/mL)	187.3 ± 34.72	585.3 ± 95.91	399.2 ± 133.6	680.6 ± 152.7
Serum ionized calcium (mmol/L)	1.12 ± 0.01	0.92 ± 0.02^b^	1.35 ± 0.01^a^^,^^d^	0.92 ± 0.05^a^^,^^g^
Serum phosphorus (mg/dL)	9.37 ± 0.39	6.49 ± 0.26^a^	6.95 ± 0.36^a^	6.96 ± 0.43^a^
Water ingestion (mL/day)	10.5 ± 1.3	24.1 ± 1.8^a^	26.8 ± 2.5^a^	25.92 ± 1.5^a^
Urine volume (mL/day)	12.79 ± 1.2	23.25 ± 1.6^a^	29.92 ± 2.6^a^	27.31 ± 2.2^a^
Urine osmolality (mOsm/kg)	1231 ± 98.33	895.6 ± 74.0^b^	516.6 ± 39.1^a^^,^^d^	608.9 ± 37.5^a^^,^^e^
Urinary protein excretion (mg/day)	20.93 ± 1.15	33.20 ± 2.15	33.33 ± 6.09	48.00 ± 6.53^b^
FECa (%)	0.72 ± 0.03	0.27 ± 0.03^a^	1.61 ± 0.13^a^^,^^d^	0.74 ± 0.07^d^^,^^g^
FEP (%)	7.68 ± 0.76	12.22 ± 1.41	27.17 ± 5.98^b^^,^^f^	26.69 ± 4.31^b^^,^^f^

C, control (standard diet); VDD, vitamin D deficiency (vitamin D-free diet); IRI, ischemia/reperfusion injury (standard diet and subjected to bilateral renal ischemia for 45 min on day 28); VDD + IRI, vitamin D deficiency + ischemia/reperfusion (vitamin D-free diet and subjected to bilateral renal ischemia for 45 min on day 28); 25(OH)D, 25-hydroxyvitamin D; InCl, inulin clearance; BW, body weight; KW/BW, kidney weight/body weight; MAP, mean arterial pressure; RVR, renal vascular resistance; PTH, parathyroid hormone; FECa, fractional excretion of calcium; FEP, fractional excretion of phosphorus. Data are mean ± SEM. ^a^*P* < 0.001, ^b^*P* < 0.01, and ^c^*P* < 0.05 versus C, ^d^*P* < 0.001, ^e^*P* < 0.01, and ^f^*P* < 0.05 versus VDD, ^g^*P* < 0.001 and ^i^*P* < 0.05 versus IRI.

### Calcium and phosphorus

Serum calcium was lower in both VDD groups than in control rats (Table1). Serum phosphorus was lower in VDD, IRI, and VDD + IRI rats than in control rats. Fractional excretion of calcium (FECa) was lower in VDD and VDD + IRI rats than in control and IRI rats, although it was higher in VDD + IRI rats than in VDD rats. In contrast, fractional excretion of phosphorus (FEP) was higher in IRI rats than in control and VDD rats. Although serum parathyroid hormone (PTH) did not differ among the groups, it trended higher in VDD, IRI and VDD + IRI rats than in control rats (Table1).

### Kidney weight

Kidneys were weighed after removal, and the kidney weight/body weight (KW/BW) ratio was calculated. As expected, 48 h after ischemia/reperfusion, KW/BW ratios were significantly higher in IRI rats than in control and VDD rats (Table1). The KW/BW ratio was highest in VDD + IRI rats, indicating greater hypertrophy/hyperplasia.

### Protein expression of PCNA, p21 and VDR

**Figure 1:**
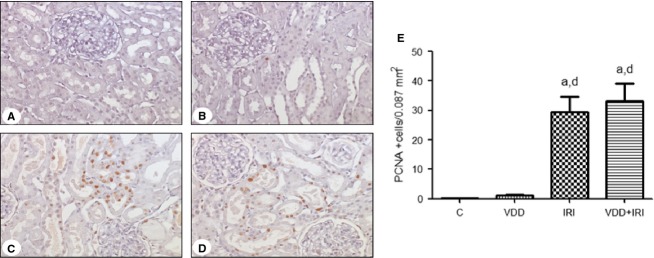
Expression of proliferating cell nuclear antigen (PCNA) in rat kidney tissue and (brown) immunostaining for PCNA in kidney samples. (A) Control group sample. (B) Vitamin D deficiency group sample. (C) Ischemia/reperfusion injury group sample. (D) Vitamin D deficiency + ischemia/reperfusion injury group sample. Magnification, ×400. (E) Bar graph of PCNA expression values. Data are mean ± SEM. C, control (standard diet); VDD, vitamin D deficiency (vitamin D-free diet); IRI, ischemia/reperfusion injury (standard diet and subjected to bilateral renal ischemia for 45 min on day 28); VDD + IRI, vitamin D deficiency + ischemia/reperfusion (vitamin D-free diet and subjected to bilateral renal ischemia for 45 min on day 28). ^a^*P* < 0.001 versus C; ^d^*P* < 0.001 versus VDD.

Immunohistochemical staining revealed that expression of PCNA (a protein involved in cell proliferation) was higher in IRI and VDD + IRI rats than in control and VDD rats (Fig.1).

**Figure 2:**
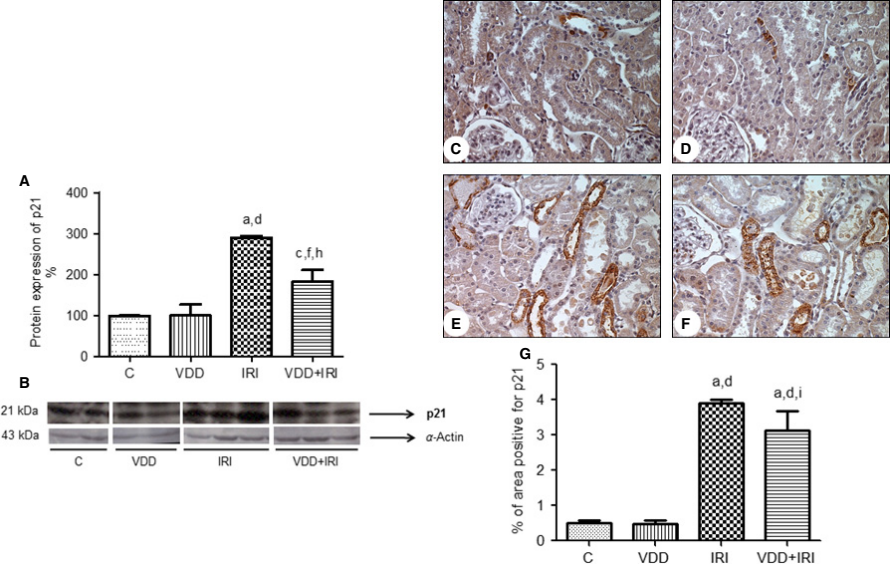
Semiquantitative immunoblotting and immunohistochemical analysis of p21 protein expression in rat kidney tissue. (A) Densitometric analysis. (B) Representative bands of immunoblots reacted with anti-p21 (revealing a 21-kDa band). (C–F) Immunostaining (brown) for p21 in kidney cortex samples from the control group (C), vitamin D deficiency group (D), ischemia/reperfusion injury group (E) and vitamin D deficiency + ischemia/reperfusion injury group (F). Magnification, ×400. (G) Bar graph of p21 protein expression values. Values are mean ± SEM. C, control (standard diet); VDD, vitamin D deficiency (vitamin D-free diet); IRI, ischemia/reperfusion injury (standard diet and subjected to bilateral renal ischemia for 45 min on day 28); VDD + IRI, vitamin D deficiency + ischemia/reperfusion (vitamin D-free diet and subjected to bilateral renal ischemia for 45 min on day 28). ^a^*P* < 0.001 versus C; ^c^*P* < 0.05 versus C; ^d^*P* < 0.001 versus VDD; ^f^*P* < 0.05 versus VDD; ^h^*P* < 0.01 versus IRI; ^i^*P* < 0.05 versus IRI.

As shown in Fig.2, p21 protein expression was significantly higher in IRI rats than in control and VDD rats (290 ± 4.8 vs. 99 ± 0.6 and 100 ± 27%; *P *<* *0.001), as well as being higher in IRI rats than in VDD + IRI rats (290 ± 4.8 vs. 182 ± 29%; *P *<* *0.01). Immunohistochemical staining confirmed these results.

**Figure 3:**
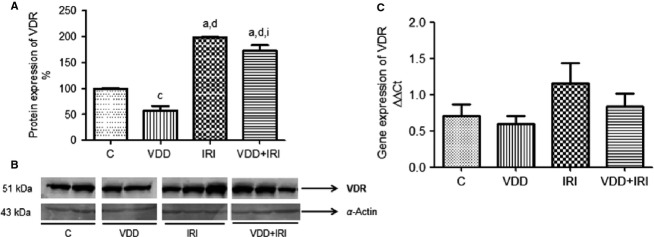
Densitometry, semiquantitative immunoblotting and real-time quantitative polymerase chain reaction analysis of vitamin D receptor expression in rat kidney tissue. (A) Densitometric analysis of all samples: control group (*n *=* *4); vitamin D deficiency group (*n *=* *5); ischemia/reperfusion injury group (*n *=* *6); vitamin D deficiency + ischemia/reperfusion injury group (*n *=* *6). (B) Representative bands of immunoblots reacted with anti-vitamin D receptor (VDR) antibody, revealing a 51-kDa band. (C) Bar graph of VDR gene expression values. Data are mean ± SEM. C, control (standard diet); VDD, vitamin D deficiency (vitamin D-free diet); IRI, ischemia/reperfusion injury (standard diet and subjected to bilateral renal ischemia for 45 min on day 28); VDD + IRI, vitamin D deficiency + ischemia/reperfusion (vitamin D-free diet and subjected to bilateral renal ischemia for 45 min on day 28). ^a^*P* < 0.001 and ^c^*P* < 0.05 versus C; ^d^*P* < 0.001 versus VDD; ^i^*P* < 0.05 versus IRI.

**Figure 4:**
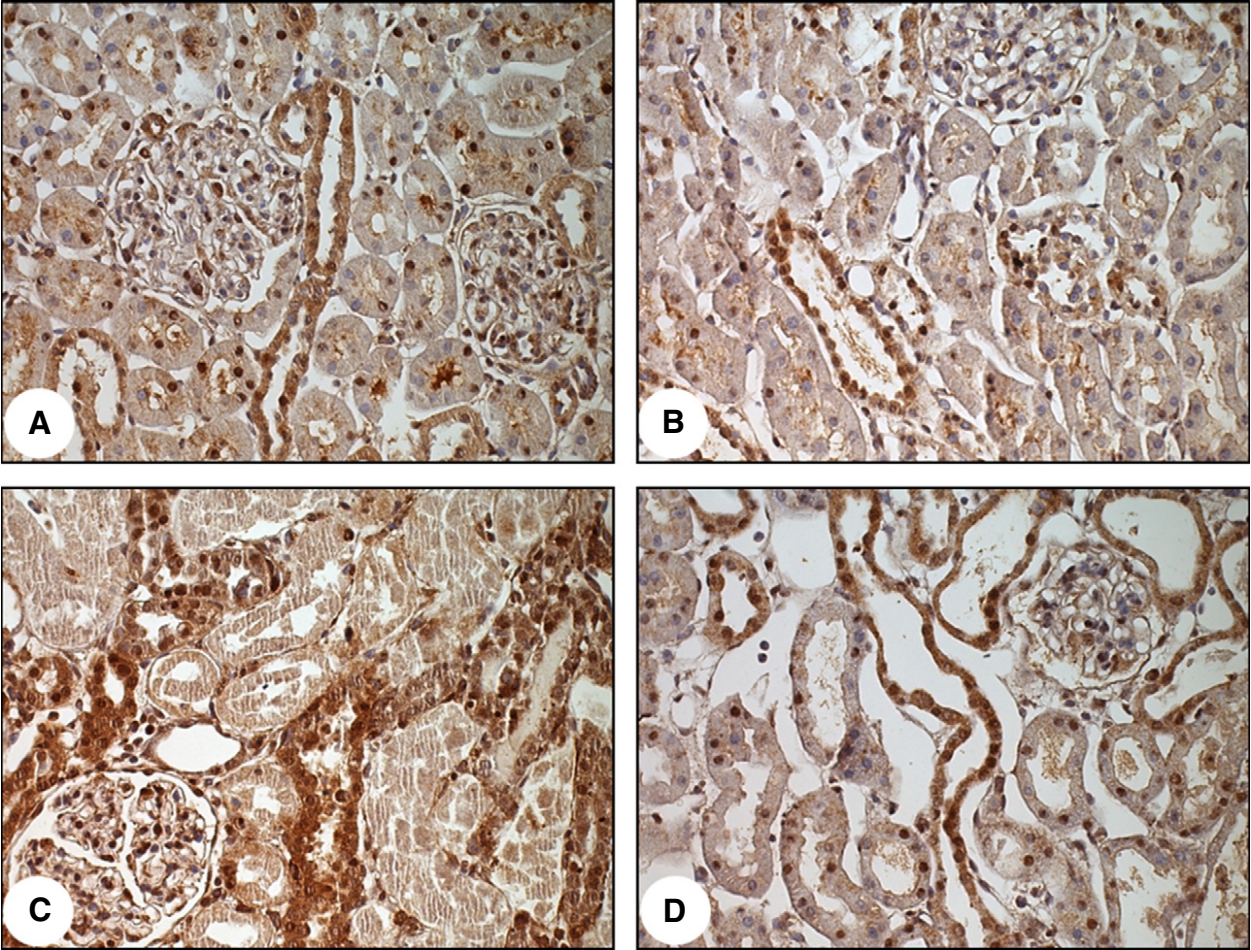
Vitamin D receptor (VDR) immunohistochemical staining in rat kidney tissue. Immunostaining (dark brown) for VDR in kidney cortex samples from the control group (A), vitamin D deficiency group (B), ischemia/reperfusion injury group (C) and vitamin D deficiency plus ischemia/reperfusion injury group (D). Magnification, ×400.

Figure3 shows that VDR protein expression was higher in IRI rats than in control and VDD rats (198 ± 0.7 vs. 99 ± 0.5 and 57.5 ± 8.5%; *P *<* *0.001), although it was lower in VDD + IRI rats than in IRI rats (172 ± 10.9 vs. 198 ± 0.7%; *P *<* *0.05). The VDD rats presented lower VDR protein expression than did the control rats. Although the difference was not significant, VDR gene expression was higher in IRI rats than in control and VDD rats, and VDR gene expression was lower in VDD + IRI rats than in IRI rats. In accordance with the gene and protein expression results, the immunohistochemical staining for VDR was more intense and extensive in IRI rats than in VDD + IRI rats. The immunolocalization of VDR was in the cytoplasm and nuclei, within the proximal tubules (Fig.4).

### Urine concentration

**Figure 5:**
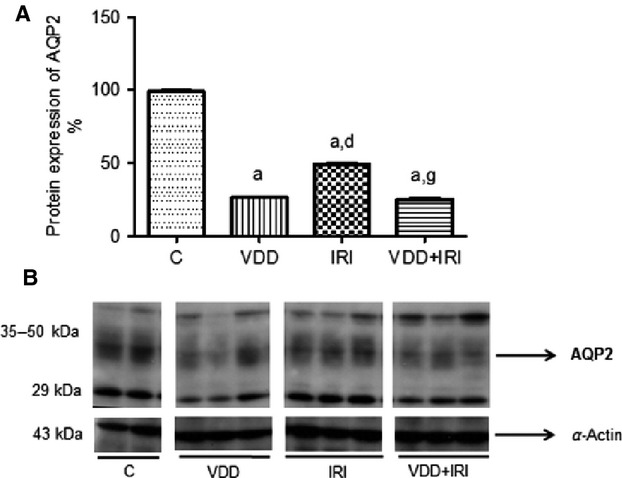
Densitometry and semiquantitative immunoblotting of AQP2 in rat kidney tissue. (A) Densitometric analysis of all samples: control group (*n *=* *4); vitamin D deficiency group (*n *=* *5); ischemia/reperfusion injury group (*n *=* *6); vitamin D deficiency + ischemia/reperfusion injury group (*n *=* *6). (B) Representative bands of immunoblots reacted with anti-aquaporin 2 antibody, revealing 29–50 kDa bands. Values are mean ± SEM. C, control (standard diet); VDD, vitamin D deficiency (vitamin D-free diet); IRI, ischemia/reperfusion injury (standard diet and subjected to bilateral renal ischemia for 45 min on day 28); VDD + IRI, vitamin D deficiency + ischemia/reperfusion (vitamin D-free diet and subjected to bilateral renal ischemia for 45 min on day 28); AQP2, aquaporin 2. ^a^*P* < 0.001 versus C; ^d^*P* < 0.001 versus VDD; ^g^*P* < 0.001 versus IRI.

**Figure 6:**
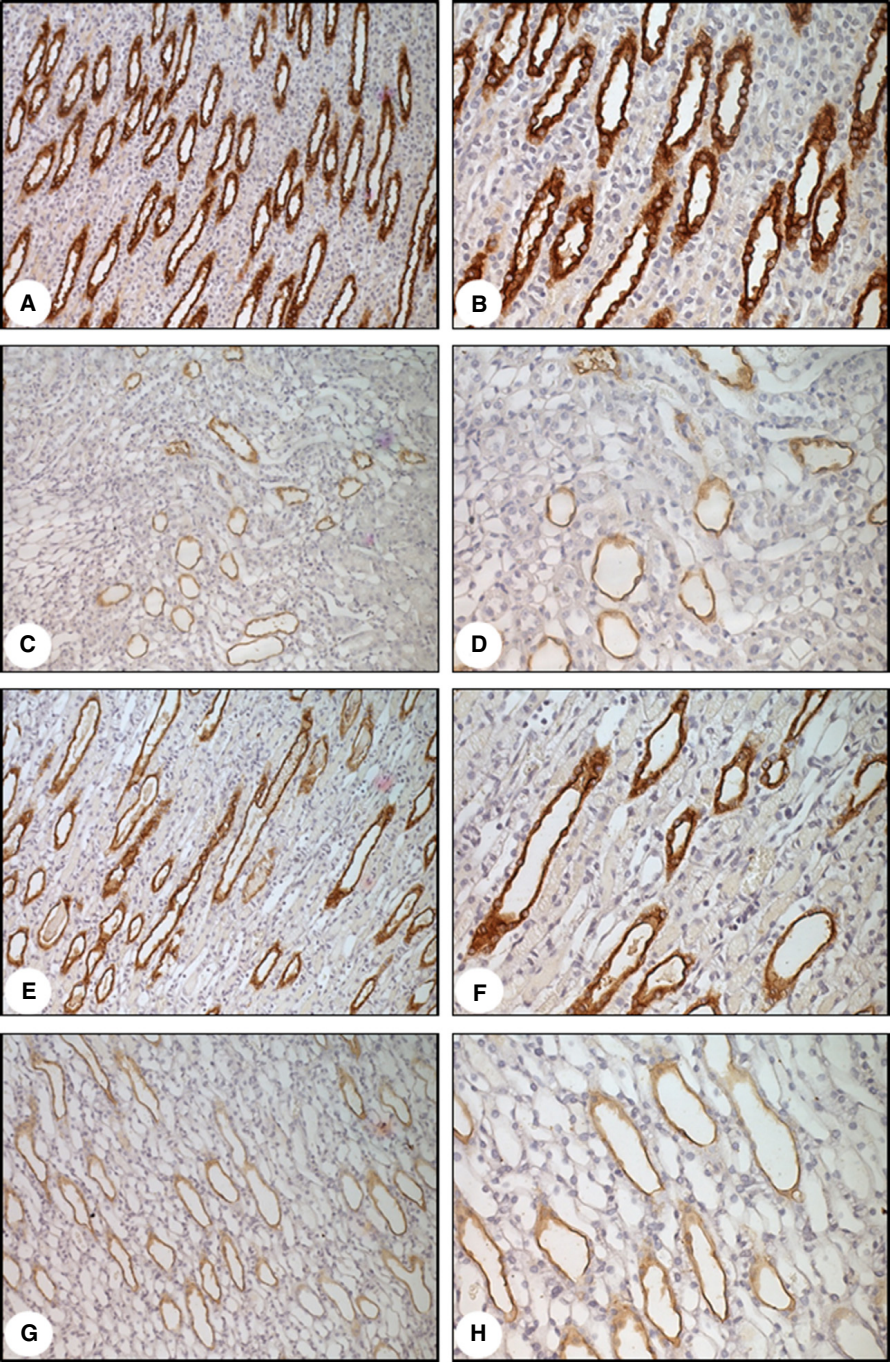
Immunostaining for aquaporin 2 in kidney medulla samples. Samples are from the control group (A and B), vitamin D deficiency group (C and D), ischemia/reperfusion injury group (E and F) and vitamin D deficiency plus ischemia/reperfusion injury group (G and H). Magnification, ×200 (in A, C, E, and G) and ×400 (in B, D, F, and H).

Compared with control rats, VDD rats showed higher urine volume and lower urine osmolality (Table1). Although urine osmolality was higher in VDD rats than in IRI and VDD + IRI rats, neither urine volume nor urine osmolality differed between the IRI and VDD + IRI rats. Semiquantitative immunoblotting (Fig.5) revealed that renal expression of aquaporin 2 (AQP2) was significantly lower in VDD rats than in control rats (26.2 ± 0.6 vs. 99 ± 1.0%; *P *<* *0.001), as well as being significantly lower in VDD + IRI rats than in IRI rats (25.2 ± 0.8 vs. 49 ± 1.0%; *P *<* *0.001). Figure6 shows representative sections of the medulla from rats in the control (A and B), VDD (C and D), IRI (E and F), and VDD + IRI (G and H) groups, probed to reveal the presence of AQP2. In the control rats, AQP2 was abundantly expressed, the comparative intensity of that expression being lower in the IRI rats and even lower in the VDD and VDD + IRI rats.

### Inflammation and tubular injury

**Figure 7:**
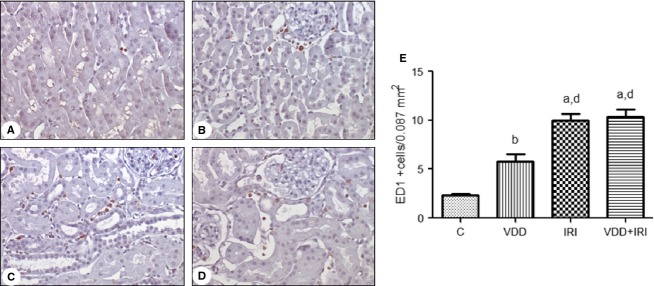
Immunohistochemical analysis of ED1 expression in rat kidney tissue. Immunostaining (brown) for ED1 in kidney cortex samples from the control group (A), vitamin D deficiency group (B), ischemia/reperfusion injury group (C) and vitamin D deficiency + ischemia/reperfusion injury group (D). Magnification, ×400. (E) Bar graph of ED1 expression values. Data are mean ± SEM. C, control (standard diet); VDD, vitamin D deficiency (vitamin D-free diet); IRI, ischemia/reperfusion injury (standard diet and subjected to bilateral renal ischemia for 45 min on day 28); VDD + IRI, vitamin D deficiency + ischemia/reperfusion (vitamin D-free diet and subjected to bilateral renal ischemia for 45 min on day 28). ^a^*P* < 0.001 and ^b^*P* < 0.01 versus C; ^d^*P* < 0.001 versus VDD.

As shown in Fig.7, the number of tubulointerstitial cells staining for ED1 (macrophages/monocytes) was significantly higher in VDD rats than in control rats (5.8 ± 0.73 vs. 2.3 ± 0.12 cells/field; *P *<* *0.01). Although there was no such difference between the IRI and VDD + IRI groups (9.98 ± 0.68 vs. 10.38 ± 0.75 cells/field), both showed significantly higher numbers of ED1+ cells than did the control and VDD groups (*P *<* *0.001 for both).

**Figure 8:**
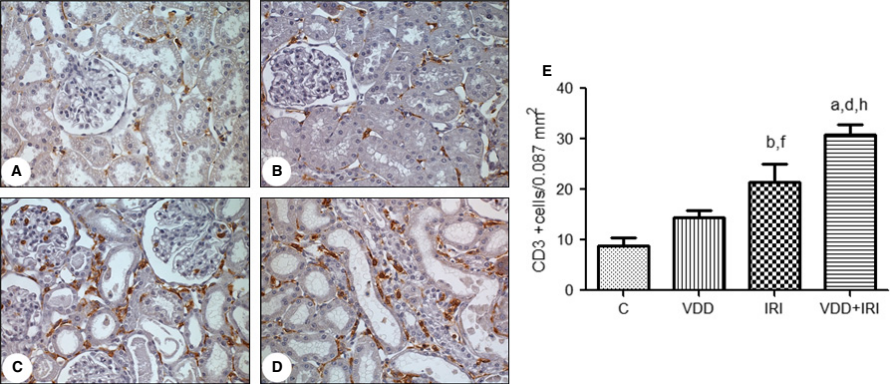
Immunohistochemical analysis of CD3 expression in rat kidney tissue. Immunostaining (brown) for CD3 in kidney cortex samples from the control group (A), vitamin D deficiency group (B), ischemia/reperfusion injury group (C) and vitamin D deficiency + ischemia/reperfusion injury group (D). Magnification, ×400. (E) Bar graph of CD3 expression values. Data are mean ± SEM. C, control (standard diet); VDD, vitamin D deficiency (vitamin D-free diet); IRI, ischemia/reperfusion injury (standard diet and subjected to bilateral renal ischemia for 45 min on day 28); VDD + IRI, vitamin D deficiency + ischemia/reperfusion (vitamin D-free diet and subjected to bilateral renal ischemia for 45 min on day 28). ^a^*P* < 0.001 and ^b^*P* < 0.01 versus C; ^d^*P* < 0.001 and ^f^*P* < 0.05 versus VDD; ^h^*P* < 0.01 versus IRI.

Figure8 shows that the number of tubulointerstitial cells staining for CD3 (lymphocytes) was significantly higher in IRI and VDD + IRI rats than in control and VDD rats (21.5 ± 3.5 and 30.9 ± 1.9 vs. 8.9 ± 1.4 and 14.5 ± 1.4 cells/field; *P *<* *0.05). Immunohistochemical analysis showed that the number of CD3+ cells was markedly higher in VDD + IRI rats than in IRI rats (*P *<* *0.01).

**Figure 9:**
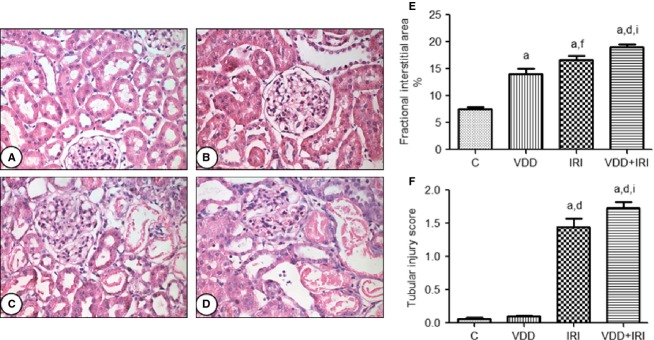
Fractional interstitial area and tubular injury in rat kidney tissue. Representative photomicrographs of kidney tissue samples from the control group (A), vitamin D deficiency group (B), ischemia/reperfusion injury group (C) and vitamin D deficiency + ischemia/reperfusion injury group (D). Magnification, ×400. (E) Bar graph of fractional interstitial area values. (F) Bar graph of tubular injury scores. Data are mean ± SEM. C, control (standard diet); VDD, vitamin D deficiency (vitamin D-free diet); IRI, ischemia/reperfusion injury (standard diet and subjected to bilateral renal ischemia for 45 min on day 28); VDD + IRI, vitamin D deficiency + ischemia/reperfusion (vitamin D-free diet and subjected to bilateral renal ischemia for 45 min on day 28). ^a^*P* < 0.001 versus C; ^d^*P* < 0.001 and ^f^*P* < 0.01 versus VDD; ^i^*P* < 0.05 versus IRI.

Figure9 shows that the fractional interstitial area (FIA) was significantly greater in VDD rats than in control rats (14.04 ± 1.02 vs. 7.5 ± 0.42%; *P *<* *0.001), as well as being significantly greater in IRI and IRI + VDD rats than in control and VDD rats. The FIA was also significantly lower in IRI rats than in VDD + IRI rats (16.7 ± 0.7 vs. 19 ± 0.49%; *P *<* *0.05). Figure7 also shows that renal tubular damage was more extensive in IRI and VDD + IRI rats than in control and VDD rats (1.44 ± 0.12 and 1.72 ± 0.09 vs. 0.06 ± 0.01 and 0.10 ± 0.01, *P *<* *0.05), although it was also significantly more severe in VDD + IRI rats than in IRI rats (*P *<* *0.05).

## Discussion

Low serum vitamin D is a known predictor of mortality in critically ill patients (Lee et al. 2009; Braun et al. 2012; Braun and Christopher 2013). Here, we showed that VDD potentiates ischemia/reperfusion-induced AKI. In our VDD + IRI rats, there was a significant decline in renal function, increased urinary protein excretion and more extensive tubular damage in comparison with our IRI rats. VDD + IRI rats also showed greater lymphocyte infiltration in renal tissue.

The pathophysiological factors responsible for postischemic renal injury remain poorly understood. Because AKI results from a multifactorial process, prevention and early treatment are the best therapeutic options (de Araujo et al. 2005). The pathogenesis of AKI involves a complex interaction among vascular, tubular and inflammatory factors, followed by a process that can restore glomerular and tubular function or by a process that results in fibrosis and progressive chronic kidney failure (Bonventre 2010; Zhang et al. 2010). There are few data on the role of VDD in decreased glomerular filtration in AKI. However, clinical studies have identified low serum vitamin D as a risk factor for AKI in critically ill patients (Braun et al. 2012; Schenk 2012; Braun and Christopher 2013). Recently, de Boer et al. (2011) suggested that low serum vitamin D is a risk factor for chronic kidney disease progression, showing that vitamin D supplementation slowed that progression.

The KW/BW ratio is a measure of cell proliferation and hypertrophy, which have been observed in models of ischemia/reperfusion (Leonard et al. 2008). Calcitriol can prevent cell damage by inhibiting cell proliferation (Holick 2013), activating the p21 gene in a VDR-dependent manner (Liu et al. 1996). In the present study, the upregulation of VDR was significantly inhibited at 48 h after ischemia/reperfusion in VDD + IRI rats, as was that of p21 expression (Figs.4). Such inhibition might play a crucial role in the pathogenesis of hyperplasia and tubular injury. Low serum vitamin D reduced calcitriol production, which can decrease VDR expression and downregulate p21. Although the VDD + IRI rats showed p21 induction that was approximately 60% of that observed in the IRI rats, the VDR protein expression in the former was approximately 85% of that observed in the latter. That suggests that another pathway is involved in blunting the p21 protein expression in VDD rats. The VDR content is not the only determinant of the efficacy of calcitriol in controlling proliferation. Therefore, the vitamin D system might involve more than a single receptor-ligand pair (Dusso et al. 2005). Importantly, micromolar concentrations of calcitriol arrest growth and induce apoptosis in mammary epithelial tumor cell lines generated from VDR-null mice, suggesting the existence of VDR-independent mechanisms (Valrance and Welsh 2004). Studies involving the parathyroid glands of 1*α*-hydroxylase-null mice have provided evidence that a novel VDR-independent mechanism mediates the antiproliferative properties of high serum calcitriol, and that calcitriol plays a critical role in controlling the growth of parathyroid cells (Panda et al. 2004).

Ischemia/reperfusion can activate cellular pathways of proliferation, inflammation, and apoptosis. AKI induces quiescent renal cells to enter to the cell cycle and activates cell cycle inhibitors, including p21, which is present in the cytoplasm. Because p21 is a potent inhibitor of cyclin activity, its increased expression can block cell cycle progression (Megyesi et al. 2001; Price et al. 2003, 2009). It has been shown that renal cell death is more widespread in mice lacking the p21 gene than in those with a functional p21 gene, as well as that the former develop more severe renal failure and have lower survival (Megyesi et al. 2001). That has been demonstrated in the ischemia/reperfusion, cisplatin and ureteral obstruction models of AKI (Megyesi et al. 1996). Tokumoto et al. (2002) showed that p21 and p27 were diminished in parathyroid glands with nodules or adenomas, also showing that parathyroid sections with high nuclear VDR expression elicited high p21 and p27 expression. Acute tubular necrosis is accompanied by increases in cell infiltration, tubular dilation and interstitial area, as well as apoptosis (de Araujo et al. 2005; Bonventre 2010), features that, in our study, were most pronounced in VDD + IRI rats, suggesting that VDD is an aggravating factor for tubular necrosis.

We found that infiltration by macrophages and T lymphocytes was greatest in VDD + IRI rats (Figs.7 and 8). This demonstrates the antiproliferative and immunomodulatory properties of vitamin D and suggests that this hormone controls renal inflammation (Osborne and Hutchinson 2002).

It is of note that MAP and RVR were statistically higher in our VDD, IRI, and VDD + IRI rats than in our control rats. Glomerular vascular resistance, driven by myogenic response, tubuloglomerular feedback, renin-angiotensin-aldosterone, nitric oxide and other substances, plays a role in renal autoregulation (Metra et al. 2012; Evans et al. 2013). VDD can lead to hypertension, involving the renin-angiotensin system, together with changes in the endothelium and vascular smooth muscle. Mice deficient in 1*α*-hydroxylase, the enzyme that converts vitamin D into its active form, calcitriol, develop hypertension and right ventricular hypertrophy (Vaidya and Williams 2012; Lucisano et al. 2013; Tamez et al. 2013).

Urinary protein excretion was significantly increased in our VDD + IRI rats. Human studies have shown that urinary protein excretion is higher in adults with VDD or vitamin D depletion than in those without (de Boer et al. 2007; Lee et al. 2011). Low serum vitamin D can increase urinary protein excretion, either directly—by inducing podocyte loss and glomerulosclerosis, thereby disrupting the integrity of the glomerular basement membrane filtration(Kuhlmann et al. 2004)—or indirectly, by suppressing renin transcription and altering the hemodynamic balance (Freundlich et al. 2008). We found that VDD-induced urinary concentrating defect. Ischemia/reperfusion is associated with a marked reduction in the renal protein expression of AQP1, AQP2, and AQP3 (Hussein et al. 2012). However, the mechanism by which vitamin D downregulates AQP2 (Figs.5 and 6), as observed here, remains unknown. In addition, the VDD group also presented a decrease in glomerular filtration and an increase in renal infiltration by macrophages/monocytes, as well as an increase in fractional interstitial area. On the basis of these findings, we conclude that VDD can cause glomerular and tubular damage. It is of note that VDR protein expression was lower in the VDD group than in the control group, although there was no difference between those two groups in terms of p21 protein expression. Another pathway might be involved in VDD-induced kidney damage. The elevated PTH levels seen in our study could be attributable to the low serum levels of calcium.

In conclusion, VDD aggravates AKI. We speculate that the way in which 25(OH)D controls renal inflammation, proliferation and cell injury in ischemic AKI is by altering p21, and VDRs might constitute one of the pathways of that mechanism. Further studies are required to determine whether correction of VDD provides clinical benefits in AKI.

## Conflict of Interest

All the authors declared no competing interests.
